# Cerebro-cerebellar circuits in autism spectrum disorder

**DOI:** 10.3389/fnins.2015.00408

**Published:** 2015-11-05

**Authors:** Anila M. D'Mello, Catherine J. Stoodley

**Affiliations:** ^1^Department of Psychology, American UniversityWashington DC, USA; ^2^Center for Behavioral Neuroscience, American UniversityWashington DC, USA

**Keywords:** autism spectrum disorder, cerebellum, neuroimaging, diffusion tensor imaging, voxel based morphometry, resting state MRI, cerebo-cerebellar circuits, functional connectivity

## Abstract

The cerebellum is one of the most consistent sites of abnormality in autism spectrum disorder (ASD) and cerebellar damage is associated with an increased risk of ASD symptoms, suggesting that cerebellar dysfunction may play a crucial role in the etiology of ASD. The cerebellum forms multiple closed-loop circuits with cerebral cortical regions that underpin movement, language, and social processing. Through these circuits, cerebellar dysfunction could impact the core ASD symptoms of social and communication deficits and repetitive and stereotyped behaviors. The emerging topography of sensorimotor, cognitive, and affective subregions in the cerebellum provides a new framework for interpreting the significance of regional cerebellar findings in ASD and their relationship to broader cerebro-cerebellar circuits. Further, recent research supports the idea that the integrity of cerebro-cerebellar loops might be important for early cortical development; disruptions in specific cerebro-cerebellar loops in ASD might impede the specialization of cortical regions involved in motor control, language, and social interaction, leading to impairments in these domains. Consistent with this concept, structural, and functional differences in sensorimotor regions of the cerebellum and sensorimotor cerebro-cerebellar circuits are associated with deficits in motor control and increased repetitive and stereotyped behaviors in ASD. Further, communication and social impairments are associated with atypical activation and structure in cerebro-cerebellar loops underpinning language and social cognition. Finally, there is converging evidence from structural, functional, and connectivity neuroimaging studies that cerebellar right Crus I/II abnormalities are related to more severe ASD impairments in all domains. We propose that cerebellar abnormalities may disrupt optimization of both structure and function in specific cerebro-cerebellar circuits in ASD.

## Introduction

Post-mortem, genetic, animal models, neuroimaging, and clinical evidence suggest that cerebellar dysfunction may play a crucial role in the etiology of autism spectrum disorder (ASD; for reviews, see Becker and Stoodley, 2013; Wang et al., 2014). The cerebellum is one of the most consistent sites of abnormality in autism (Allen, 2005; Fatemi et al., 2012), with differences reported from the cellular to the behavioral level. The majority of post-mortem studies of ASD report decreased Purkinje cell counts in the cerebellar cortex (Fatemi et al., 2002; Bauman and Kemper, 2005), and ASD-like symptoms can be induced by specifically targeting cerebellar Purkinje cells in animal models (Tsai et al., 2012). Cerebellar structural differences are associated with social and communication impairments as well as restricted interests and repetitive behaviors, the hallmarks of the ASD diagnosis, in both human studies (Pierce and Courchesne, 2001; Rojas et al., 2006; Riva et al., 2013; D'Mello et al., 2015) and animal models of ASD (Ingram et al., 2000; Brielmaier et al., 2012; Tsai et al., 2012). The cerebellar cortex was consistently abnormal in an analysis of over 26 mouse models of ASD (Ellegood et al., 2015), and cerebellar atrophy is characteristic of one of the most widely used animal models of ASD, the valproic acid model (Ingram et al., 2000). At the genetic level, genes implicated in ASD (e.g., SHANK3, EN2, RORA) are often involved in cerebellar development (see Rogers et al., 2013 for review). This suggests that cerebellar development may be disrupted in ASD, which could have major knock-on effects on the structure and function of the multiple regions of the cerebral cortex with which the cerebellum forms reciprocal connections (see Wang et al., 2014; for reviews, see Strick et al., 2009; Stoodley and Schmahmann, 2010; Buckner et al., 2011).

The cerebellum is interconnected with distributed regions of the cerebral cortex, including regions involved in sensation (e.g., Snider and Stowell, 1944), movement (e.g., Snider and Eldred, 1951), attention (e.g., Kellermann et al., 2012), reward/motivation (e.g., Snider and Maiti, 1976), language (e.g., Schmahmann and Pandya, 1997; Kelly and Strick, 2003; Booth et al., 2007; Strick et al., 2009), social processing (e.g., Jissendi et al., 2008; Sokolov et al., 2012; Jack and Pelphrey, 2014), memory (e.g., Heath and Harper, 1974), and executive function (e.g., Middleton and Strick, 2000; Habas et al., 2009). This extensive connectivity provides an anatomical substrate by which cerebellar dysfunction could be involved in the large spectrum of symptoms that comprise the autism diagnosis (Rogers et al., 2013). We hypothesize that disruptions in *specific* cerebro-cerebellar loops in ASD might impede the functional and structural specialization of cortical regions involved in motor control, language, and social interaction, leading to developmental impairments in these domains. Here, after providing background information about cerebellar topography and cerebro-cerebellar circuits, we discuss the potential importance of the cerebellum in development, and review structural and functional neuroimaging studies describing regional cerebellar differences and disrupted cerebro-cerebellar circuits in ASD. We frame these findings in the context of the broader cerebro-cerebellar circuits involved in movement, language, and social cognition. We then address potential mechanisms by which cerebellar dysfunction could impact the core behavioral features of ASD. Finally, we suggest future directions for research.

## Cerebellar topography and cerebro-cerebellar circuits

**Figure 1:**
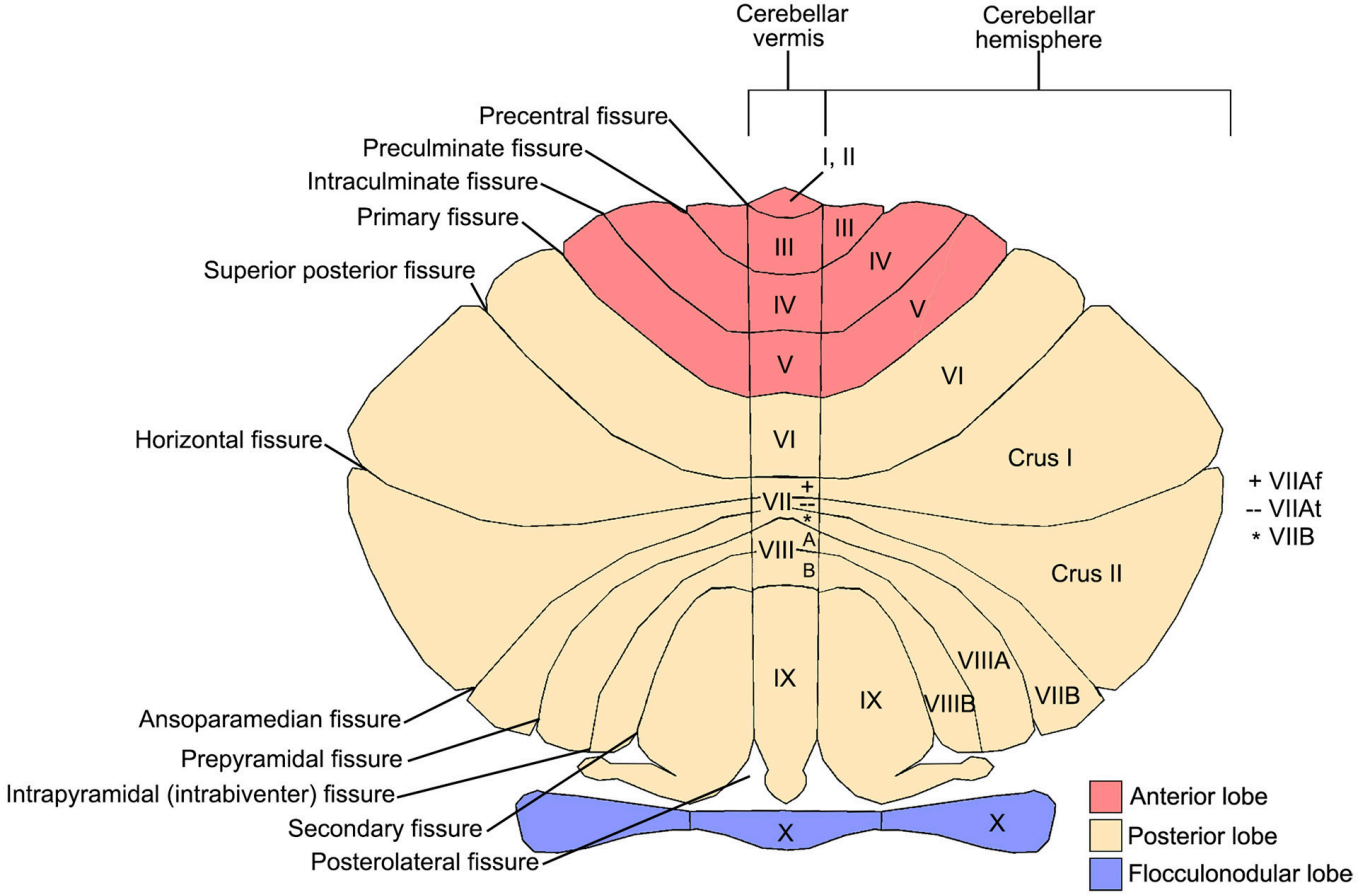
**Cerebellar anatomy showing major fissures, lobes, and lobules**. The cerebellum is flattened to show the anterior lobe (red; lobules I-V), posterior lobe (cream; lobules VI-IX), and flocculonodular lobe (purple; lobule X). The 10 cerebellar lobules (labeled I-X) are labeled in both the vermis and hemispheres. Lobule VII is subdivided into Crus I, Crus II, and VIIB in the hemispheres, and VIIAf, VIIAt, and VIIB in the vermis. Lobule VIII is subdivided into VIIIA and VIIIB. Figure courtesy of Professor Jeremy Schmahmann, Massachusetts General Hospital, Boston.

**Figure 2:**
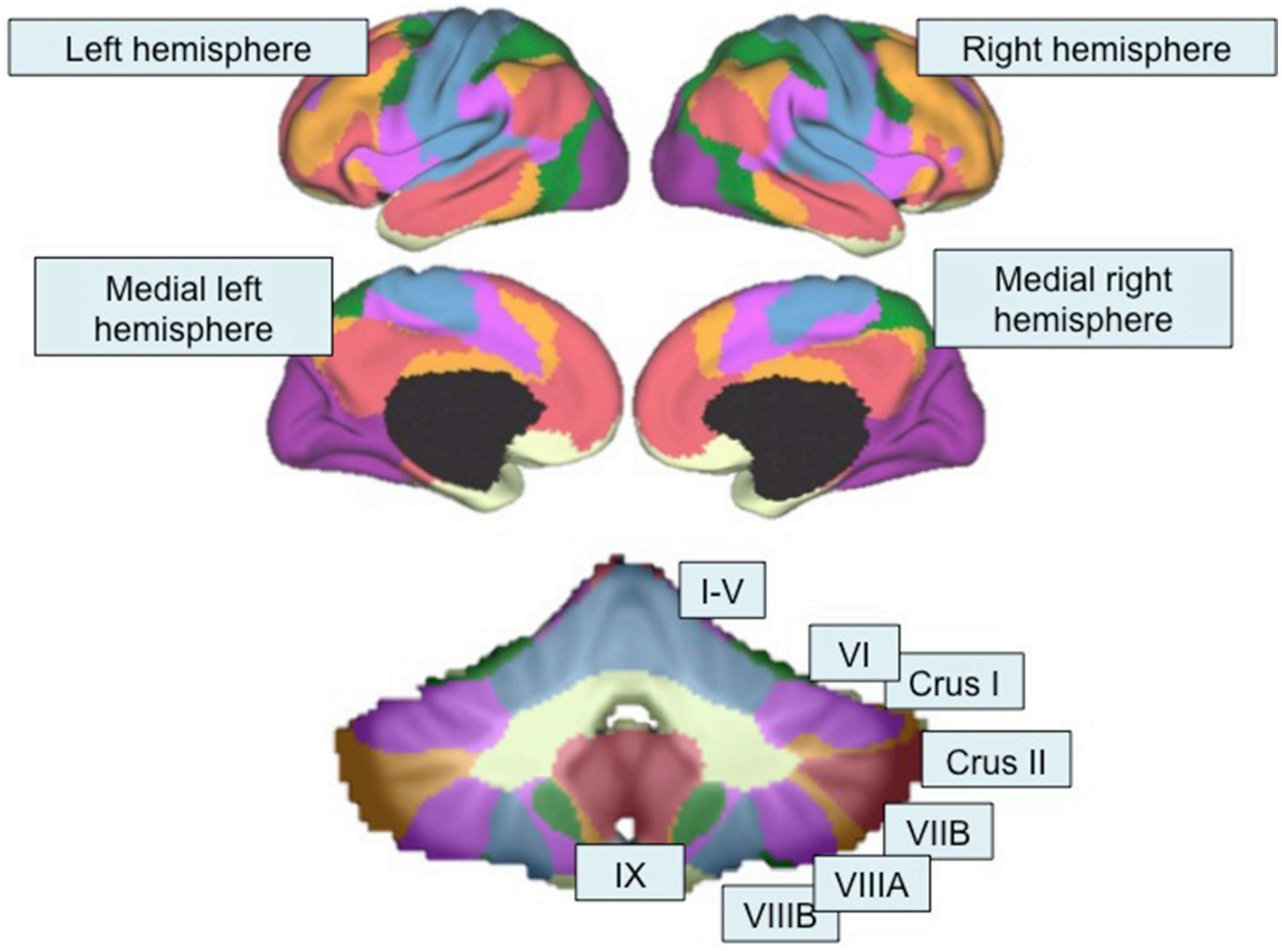
**Functional topography revealed by resting-state functional connectivity mapping**. Top, Color-coded seven-network map of the cerebral cortex as revealed by resting-state functional connectivity (adapted with permission from Yeo et al., 2011). Bottom, resting-state functional connectivity network map of the cerebellum using the same seven-network solution (Buckner et al., 2011) from the Spatially Unbiased Infratentorial (SUIT) Atlas (Diedrichsen, 2006; Diedrichsen et al., 2009). Lobules are labeled according to the scheme shown in Figure 1. Purple, visual network; blue, somatomotor network; green, dorsal attention network; violet, ventral attention network; cream, limbic network; orange, fronto-parietal network; red, default mode network.

The emerging topography of sensorimotor, cognitive, and affective subregions in the cerebellum provides an important framework for interpreting the functional significance of cerebellar findings in ASD and their relationship with broader cerebro-cerebellar circuits. The cerebellum forms reciprocal, closed-loop circuits with much of the cerebral cortex as well as subcortical structures; because of this closed-loop organization and uniform circuitry, it is thought that the cerebellum contains repeating processing modules, the function of which is driven by the input the module receives (Schmahmann, 1991; Ito, 2006). Therefore, functional subregions of the cerebellum exist because different regions of the cerebellum form circuits with specific regions of the cerebral cortex. The anterior cerebellum is structurally and functionally connected to sensorimotor areas of the cerebral cortex, while the posterior cerebellum is structurally and functionally connected to “cognitive” regions, including prefrontal, and parietal association cortices (Strick et al., 2009; Stoodley and Schmahmann, 2010; Buckner et al., 2011; see Figures 1, 2). The cerebellar deep nuclei—which receive projections from the cerebellar cortex and send output fibers from the cerebellum—also mirror this functional topography. In particular, the large dentate nuclei can be separated into dorsal and ventral regions that project to non-motor and motor regions of the cerebral cortex, respectively (Dum and Strick, 2003; Küper et al., 2011). This cerebellar functional topography is robust and is evident even at the individual level (Stoodley et al., 2010).

The specific cerebro-cerebellar circuits described above are involved in different aspects of behavior. In clinical studies, the location and lateralization of cerebellar damage can predict the resulting symptomology. Damage to the anterior cerebellum can result in motor symptoms such as ataxia (Schmahmann et al., 2009), while posterior lobe damage can lead to cognitive impairments and affective dysregulation (the Cerebellar Cognitive Affective Syndrome; Schmahmann and Sherman, 1998). Posterior vermal tumor resection has been associated with behavioral disturbances and affective dysregulation (Levisohn et al., 2000; Riva and Giorgi, 2000), reflecting anatomical connections between the vermis and limbic areas (Stoodley and Schmahmann, 2010; Buckner et al., 2011). Deficits following cerebellar damage can reflect the largely contralateral cerebro-cerebellar projections, as right cerebellar hemispheres interconnect with left cerebral language regions, and left cerebellar hemispheres form circuits with right cerebral cortical regions involved in spatial processing. Consistent with these projections, damage to the right cerebellar hemisphere in children has been associated with reduced verbal and literacy skills (Riva and Giorgi, 2000; Scott et al., 2001; Bolduc and Limperopoulos, 2009; Bolduc et al., 2012), while left cerebellar damage has been associated with impaired non-verbal or spatial skills (Riva and Giorgi, 2000; Scott et al., 2001). Lastly, lesions and neuromodulation of the cerebellum alter neural activity in regions of the cerebral cortex to which the cerebellum projects (e.g., Galea et al., 2011; Adamaszek et al., 2015), reflecting the functional impact of these long-range cerebro-cerebellar projections.

## The cerebellum, the developing brain, and neurodevelopmental disorders

Cerebellar structural and functional differences are found in several neurodevelopmental disorders, including attention deficit hyperactivity disorder (ADHD) and developmental dyslexia as well as ASD; it is important to note that different regions of the cerebellum show structural differences in each of these disorders, suggesting different cerebro-cerebellar circuits may be affected in ASD, ADHD, and dyslexia (see Stoodley, 2014). Other neurodevelopmental disorders, such as developmental coordination disorder (DCD), frequently co-occur with ADHD and dyslexia and are also hypothesized to be a product of cerebellar dysfunction (Zwicker et al., 2011; Biotteau et al., 2015). Why might cerebellar dysfunction be involved in so many developmental disorders, and what can we learn from the localization of cerebellar differences in each disorder? Relative to other regions of the brain, the cerebellum undergoes enormous growth between 24 and 40 weeks post-conception, increasing approximately 5-fold in volume and over 30-fold in surface area (see Volpe, 2009 for review). While this rapid cerebellar growth slows postnatally, neural differentiation and growth of axonal inputs and outputs continue throughout the first postnatal year (Volpe, 2009). This substantial prenatal growth, continued postnatally, might render the cerebellum especially vulnerable to developmental disruptions and damage. Consistent with this, premature infants for whom this rapid cerebellar development is interrupted are at increased risk of cerebellar hemorrhages and future neurodevelopmental disabilities (Volpe, 2009). As mentioned above, cerebellar damage is associated with a range of long-term motor, cognitive and affective outcomes, and cerebellar injury in childhood can often result in poorer outcomes than cerebellar damage in adulthood (Scott et al., 2001; Wang et al., 2014). This is evident in the assessment of acquired ASD symptoms: while damage to the cerebral cortex early in life does not lead to long-term ASD symptoms or diagnoses (Wang et al., 2014), early cerebellar injury results in an increased risk of internalizing behavioral problems, withdrawal from social contact, and affective and attentional deficits (e.g., Limperopoulos et al., 2007). Following cerebellar tumor resection, children are at an unusually elevated risk for cognitive and adaptive impairments (Beebe et al., 2005) and damage to the vermis can lead to long-term affective dysregulation (Levisohn et al., 2000). Malformations of the vermis are also associated with higher rates of affective and behavioral deficits, including ASD symptomology (Tavano et al., 2007).

More specifically, congenital cerebellar malformations and a variety of early cerebellar lesions have been directly associated with ASD diagnoses. In fact, Schmahmann included “autism spectrum” amongst the clinical characteristics of psychiatric outcomes associated with cerebellar damage or disease (Schmahmann et al., 2007). Damage to the cerebellum in infancy is one of the highest risk factors for developing ASD (estimated 40-fold increase; Limperopoulos et al., 2007), second only to having an identical twin with autism, and conferring a larger risk than having a sibling with ASD (Wang et al., 2014). In children with tuberous sclerosis, tuber load in the cerebellum was a specific predictor of ASD (Weber et al., 2000). In one pediatric case, cerebellar damage led to stereotyped movements, gaze aversion, linguistic impairments, and a complete avoidance of physical contact, ultimately resulting in an ASD diagnosis (Riva and Giorgi, 2000).

These data from clinical disorders and acquired cerebellar damage suggest that disrupted cerebellar processing has long-term effects in developmental populations, including increases in ASD diagnoses. It has been proposed that early cerebellar damage impacts the development of cerebral cortical regions to which the cerebellum projects, via “developmental diaschisis” (Wang et al., 2014). Therefore, cerebellar developmental differences in ASD could disrupt not only cerebellar function, but also could negatively impact the structure and function of multiple regions of the cerebral cortex to which the cerebellum projects.

## Are specific cerebro-cerebellar pathways disrupted in ASD? evidence from structural and functional connectivity studies

Reduced number and microstructural integrity of cerebellar fibers might disrupt the outflow pathways from the cerebellum to supratentorial regions important for movement, language, cognition, and social interaction. White matter (WM) abnormalities in the cerebellum have been consistently reported in ASD using a variety of analysis methods, including voxel-based morphometry (McAlonan et al., 2008; Sahyoun et al., 2010). More specific analysis of fiber tracts within the cerebellum and its input and output pathways have utilized diffusion tensor imaging (DTI) and tractography methods. Measurements of fractional anisotropy (FA) represent diffusion of water molecules within an axon; higher levels of FA are typically related to increased microstructural integrity or fiber organization, while reduced myelination, inflammation along the axon, and decreased fiber density or coherence might result in decreased FA. Measures of mean diffusivity (MD) are related to the interstitial space between gray and white matter, and higher MD values might reflect reduced number of neural and glial cells or reduced packing of these cells (Beaulieu, 2002). While DTI findings in ASD are not always consistent, multiple studies report decreased FA and increased MD in the corpus callosum, cingulum, and WM within the temporal and frontal lobes (Travers et al., 2012). While fewer studies have examined the cerebellum, individuals with ASD display abnormalities in structural connectivity both within the cerebellum and in the projection fibers carrying information to and from the cerebellum.

Within the cerebellum, decreases in FA and increases in MD might result from reductions in Purkinje cell size and number (Fatemi et al., 2002; Bauman and Kemper, 2005), as well as increased inflammation and microglial activation (Vargas et al., 2005), both of which are well-documented in ASD. Cerebellar WM is an especially potent discriminator of ASD diagnosis: One study in preschool children reported that increased cerebellar WM was the strongest discriminator of future ASD diagnosis, and including cerebellar WM in the model led to a correct diagnosis in 95.8% cases (Akshoomoff et al., 2004). Cerebellar WM differences may be directly related to genetic factors in ASD, as only twins concordant for the ASD phenotype had concordant cerebellar WM volumes, while twins discordant for the ASD phenotype did not have similar cerebellar WM volumes (Kates et al., 2004).

The output and input pathways of the cerebellum also show differences in FA and MD in ASD. FA and MD abnormalities in the middle cerebellar peduncle (MCP) and inferior cerebellar peduncle (ICP) might affect the relay of information to the cerebellum from the cerebral cortex and the spinal cord/inferior olives/vestibular nuclei, respectively; structural differences in the superior cerebellar peduncle (SCP), the major efferent WM tract from the cerebellum to the cerebral cortex, could reflect disruption in pathways exiting the cerebellum. In particular, 6 out of 7 studies reporting abnormalities in the cerebellar peduncles in ASD found differences in the MCP (Brito et al., 2009; Cheng et al., 2010; Shukla et al., 2010; Sivaswamy et al., 2010; Groen et al., 2011; Hanaie et al., 2013), with fewer reports of differences in the SCP (Catani et al., 2008; Brito et al., 2009; Sivaswamy et al., 2010). Most of these reported decreased FA and increased MD (Brito et al., 2009; Shukla et al., 2010; Groen et al., 2011; Hanaie et al., 2013). Less often, *increased* FA was reported in the MCP (Cheng et al., 2010; Sivaswamy et al., 2010).

Reversals in FA lateralization patterns in the cerebellar peduncles are also associated with ASD. One study found a reversed pattern of FA asymmetry in the MCP and the ICP: Typically-developing children displayed higher FA in the left MCP, while children with ASD displayed the opposite pattern, with higher FA in the right MCP. This is consistent with lateralization differences seen in structural and functional imaging studies, whereby individuals with ASD show abnormal rightward lateralization in cerebral cortex (e.g., Dawson et al., 1982; Escalante-Mead et al., 2003; Takeuchi et al., 2004; Flagg et al., 2005; Knaus et al., 2010; Lindell and Hudry, 2013; Seery et al., 2013), which may continue through the MCP into the cerebellum, and within the cerebellum itself. A similar pattern was noted in the ICP: children with ASD had lower FA in the right ICP while their typically-developing counterparts displayed higher FA in the right ICP relative to the left (Sivaswamy et al., 2010).

Structural abnormalities in both the MCP and SCP imply disruption in the entire cerebro-cerebellar loop in ASD, from the cerebral cortex to the cerebellar cortex and back again. Decreased integrity of cerebellar outflow pathways might result in loss of modulatory input from the cerebellum to cortical regions involved in motor behavior and social processing. Behavioral evidence supports this, as decreased FA in the right and left SCPs were related to both increased repetitive behaviors and social impairments in ASD, respectively (Catani et al., 2008; Hanaie et al., 2013).

More specific investigations have shown that the cerebellar projections to the thalamus (which would then project to the cerebral cortex) are abnormal in ASD. In young ASD children (under 5 years of age), reduced FA was found in connections between the dentate nucleus and thalamus. Reduced FA in projections from the right *ventral* dentate to the thalamus correlated with more severe communication impairments in ASD, while reduced FA in projections from the right *dorsal* dentate to the thalamus showed a trend-level correlation with daily living skills (Jeong et al., 2012). Correlations between reduced FA in right ventral dentate nucleus projections and impaired communication in ASD might reflect disruption in cerebro-cerebellar loops between cognitive regions of the cerebellum and contralateral supratentorial language regions via the thalamus. On the other hand, reduced FA in efferents originating in the dorsal dentate nucleus and passing via the contralateral thalamus to motor cortices might impair daily living skills in which motor behavior is particularly important.

These findings of altered structural integrity of cerebro-cerebellar loops in ASD converge with the results of functional connectivity studies. Functional connectivity (FC) provides a measure of the correlation between distinct brain regions based on low-frequency fluctuations in the blood-oxygen level dependent (BOLD) signal. Resting state FC (rsFC) is acquired in the absence of any task and can provide insight into the intrinsic organization of the brain, while task-based FC can provide important information regarding network integrity during a task and can be related to task performance. In general, FC findings in ASD suggest that cerebro-cerebellar networks are poorly assembled, with both decreased connectivity within established networks and increased out-of-network patterns of connectivity (Noonan et al., 2009; Khan et al., 2015). Consistent with atypical lateralization in the peduncles, lateralization of functional connectivity patterns is abnormal in ASD. Children with ASD have increased functional connectivity between right hemisphere cerebral cortical regions and right hemisphere cerebellar regions, violating typical patterns of contralateral cerebro-cerebellar connectivity (Noonan et al., 2009; Khan et al., 2015).

Recent functional connectivity analyses in ASD suggest that the cerebellum is abnormally connected with both motor and non-motor regions of the cerebral cortex. For example, while the typically-developing group showed FC between the right cerebellum and left cerebral cortical areas, ASD participants showed atypical, additional FC between the right cerebellum and the right-hemisphere homologs of those regions (Noonan et al., 2009). This “extra” functional connectivity between regions that are not typically correlated often occurs outside of topographical principles of cerebellar organization. For example, the expected cerebro-cerebellar connectivity between left lobule VI and the middle frontal gyrus was noted in both typically-developing and ASD groups, but only the ASD participants had *additional* atypical connectivity between the left middle frontal gyrus and the right anterior cerebellum (lobules IV/V, which usually show connectivity with somatomotor networks) (Noonan et al., 2009). This recruitment of additional or “non-canonical” cerebellar regions is found in both studies examining cerebro-cerebellar FC in ASD (Noonan et al., 2009; Khan et al., 2015). Children and adolescents with ASD displayed increased rsFC between non-motor areas of the cerebellum (lobules VI and Crus I) and sensorimotor cerebral cortical regions, such as the premotor/primary motor cortices, primary somatosensory cortex, and the occipital lobe (Khan et al., 2015). This increase in non-canonical rsFC with posterolateral cerebellar regions in ASD is also evident in task-based fMRI: During simple motor tasks, individuals with ASD activate posterior cerebellar regions in addition to the anterior cerebellar regions typically recruited (Müller et al., 2003; Allen et al., 2004). These findings suggest that, during simple motor tasks, the domain specificity of cerebro-cerebellar connections might be abnormal in ASD, and may reflect the reduced integrity and abnormal organization of WM pathways entering and leaving the cerebellum.

**Figure 3:**
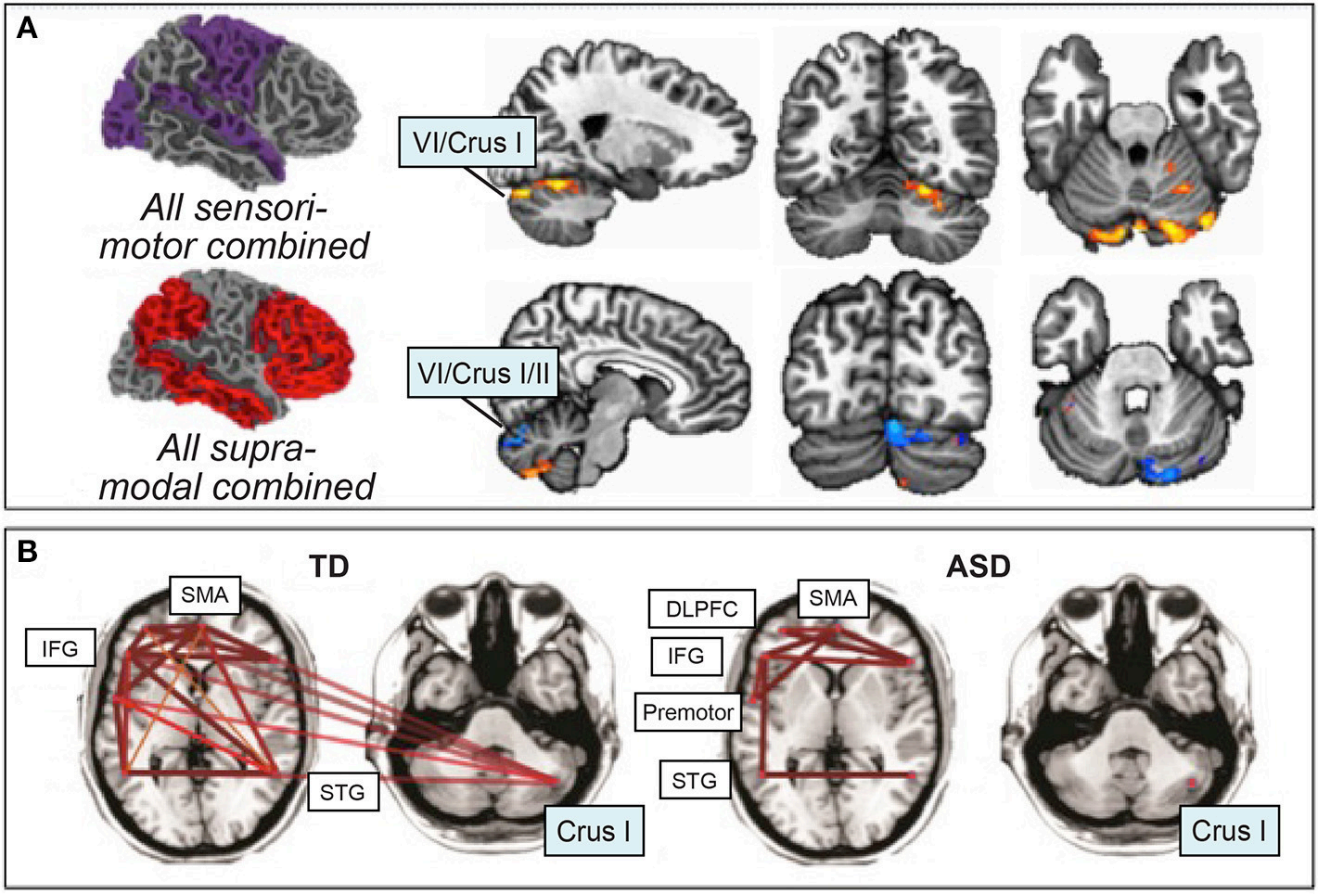
**Resting-state functional connectivity in ASD**. **(A)** Atypical increased functional connectivity between sensorimotor regions of the cerebral cortex and cerebellar lobules VI and VII (orange), decreased functional connectivity between supra-modal association cortices and lobules VI and VII (blue). Orange, ASD greater rsFC than typically-developing; blue, ASD less rsFC than typically-developing. Figure adapted with permission from Khan et al. (2015). **(B)** Preserved functional connectivity in ASD between supratentorial language regions, in contrast with the lack of cerebro-cerebellar connectivity between right Crus I/II and left-hemisphere language regions. Figure adapted from Verly et al. (2014).

This increased functional connectivity between unexpected, non-canonical regions in ASD is accompanied by decreased typical (or canonical) connectivity, particularly in cerebro-cerebellar networks related to language and social interaction (see Figure 3). Compared to their typically-developing counterparts, ASD children and adolescents display reduced rsFC between right Crus I/II and contralateral prefrontal cortex, posterior parietal cortex, and the inferior/middle temporal gyrus (Khan et al., 2015). Similarly, reductions in rsFC between right Crus I and the contralateral superior frontal gyrus, middle frontal gyrus, thalamus, anterior cingulate gyrus, and parietal areas were found in ASD adolescents (Verly et al., 2014). In this study, reduced rsFC was also found with SMA and precentral gyrus (Verly et al., 2014), which is not consistent with the other studies reporting increased non-canonical FC between right Crus I/II and motor regions of the cerebral cortex in ASD described above (Khan et al., 2015).

These findings suggest that increases in resting-state cerebro-cerebellar connectivity in ASD might be primarily driven by atypical functional connectivity, particularly between lobules VI and VII (Crus I and II) and motor cortices. These increases in non-canonical connectivity might occur at the expense of canonical rsFC between the posterior cerebellum (Crus I and II) and cerebral cortical regions involved in language and social cognition, as evidenced by consistent FC decreases in these specific pathways. Indeed, such connectivity differences are associated with more impaired behaviors in ASD. Reduced connectivity between right Crus I/II and prefrontal regions such as the dorsolateral and medial prefrontal cortex correlated with increasing ASD symptoms and severity (Jung et al., 2014; Verly et al., 2014). In exploratory analyses, cerebellar connectivity with left sensorimotor and association cortices correlated with Social Responsiveness Scale (SRS) scores in ASD (Khan et al., 2015). Therefore, together with the structural data described above, these findings suggest that alterations in cerebro-cerebellar functional connectivity are related to symptom severity in ASD.

## Cerebro-cerebellar circuits and core ASD symptoms: sensorimotor, language/communication, and social interaction

Cerebellar structural and functional neuroimaging findings in ASD conform to the principles of cerebellar functional topography and can be interpreted in the context of cerebro-cerebellar circuits. Below, we consider regional cerebellar findings from structural and functional imaging studies, as well as data emerging from investigations of cerebro-cerebellar circuits using structural and functional connectivity methods, in relation to sensorimotor, language, and social interaction deficits in ASD. Throughout, when data are available, we discuss how these findings relate to core ASD symptoms.

### The sensorimotor cerebellum and sensorimotor cerebro-cerebellar circuits in ASD

The anterior cerebellum (lobules I-V) forms reciprocal loops with sensorimotor regions of the cerebral cortex, including the primary motor cortex (Strick et al., 2009), supplementary motor area and premotor cortices (Strick et al., 2009), and the basal ganglia (Bostan and Strick, 2010). The cerebellum contains multiple homunculi, including a somatotopic representation of the body in the anterior lobe extending into lobule VI, and secondary representations in lobule VIII of the posterior lobe, which also interconnects with somatomotor networks (Snider, 1950; Grodd et al., 2001; Buckner et al., 2011).

Regional structural and functional findings in ASD can be correlated with performance on motor measures and interpreted in the context of these cerebro-cerebellar loops. Decreased gray matter (GM) in the anterior cerebellum (lobules IV and V) and lobule VIII have been found to correlate with increased severity of repetitive and stereotyped behaviors (Rojas et al., 2006; D'Mello et al., 2015). In typically-developing individuals, these regions of the cerebellum are strongly activated during simple motor tasks such as finger-tapping (Stoodley and Schmahmann, 2009; Stoodley et al., 2012). However, ASD individuals showed hypoactivation in the anterior cerebellum during motor tasks when compared to age-matched controls (Müller et al., 2003; Mostofsky et al., 2009; Murphy et al., 2014), even in the context of similar engagement of primary motor cortex in both groups (Mostofsky et al., 2009). Hypoactivation in the anterior cerebellum was also related to increased number of errors and slower reaction times relative to typically-developing counterparts (Müller et al., 2001, 2003; Murphy et al., 2014). These reductions in activation also extend to task-based functional connectivity: for example, during finger tapping, individuals with ASD had decreased FC between the anterior cerebellum and primary motor cortex, thalamus, and supplementary motor area (Mostofsky et al., 2009).

Other studies report *increased* activation in the anterior cerebellum during simple motor tasks in ASD (Allen and Courchesne, 2003; Allen et al., 2004). Increased anterior lobe activation in these studies was often accompanied by more diffuse cerebellar activation, which spread into the contralateral anterior lobe as well as posterior lobe regions not typically activated during motor tasks (Allen and Courchesne, 2003; Allen et al., 2004). However, increased activation was not associated with significant behavioral differences in motor performance between ASD and typically-developing groups (Allen and Courchesne, 2003; Allen et al., 2004). In typically-developing individuals, anterior lobe activation during finger-tapping is ipsilateral to the hand being moved and does not extend into Crus I/II or contralateral anterior lobe regions (Desmond et al., 1997; Stoodley et al., 2012). Activation in posterolateral regions of the cerebellum therefore might reflect the abnormal functional circuitry between the “non-motor” cerebellum and motor areas that has been reported in ASD (Khan et al., 2015). Alternatively, decreased anterior lobe activation in ASD might be related to behavioral impairments during motor tasks, while increased anterior lobe activation might be a compensatory mechanism, allowing ASD individuals to maintain typical levels of performance.

Posterior lobules of the cerebellum such as Crus I/II *are* activated in typically-developing individuals during more complex motor paradigms, particularly during motor imitation (Jack et al., 2011; Jack and Pelphrey, 2014), and motor imitation paradigms are associated with reduced activation in right Crus I in ASD (Jack and Morris, 2014). Some have suggested that impairments in imitation and praxis are core deficits in ASD and might contribute to social and communication impairments (Rogers and Pennington, 1991; Mostofsky et al., 2006). Supporting this, in typically-developing individuals, right Crus I/II typically interconnects with frontal and parietal association areas, including areas important for processing biological motion (e.g., superior temporal sulcus; Jack et al., 2011; Sokolov et al., 2012). Consistent with this, during a more complex motor imitation paradigm, adolescents with ASD had decreased connectivity between right Crus I and the superior temporal sulcus (STS) (Jack and Morris, 2014).

Differences in WM structure in motor regions of the cerebellum have also been reported in ASD. Consistent with motor symptoms being some of the earliest signs of ASD, young children with ASD had increased MD in the anterior cerebellum and lobule VIII (Walker et al., 2012). Further, decreased FA in bilateral lobule VIII has been correlated with increased repetitive behaviors (Cheung et al., 2009). As noted above, lobule VIII is activated by motor tasks and related to motor processing in typically-developing adults, and reduced GM in this region is associated with increased repetitive behaviors in ASD (Rojas et al., 2006; D'Mello et al., 2015). These behavioral correlates of WM abnormalities in ASD suggest that cerebellar structural differences have predictable behavioral consequences on stereotyped and repetitive behaviors.

Decreased GM in the posterior cerebellar vermis (vermal lobules VI-VII) and right Crus I have also been associated with increased repetitive behaviors and stereotyped interests (Pierce and Courchesne, 2001; D'Mello et al., 2015). While these posterior areas are typically considered part of cognitive control networks, it has been suggested that repetitive behaviors in ASD might reflect a loss of cognitive control over motor areas (e.g., Mosconi et al., 2009). There are anatomical links between Crus II/VIIB of the cerebellum and both associative (with input from prefrontal cortex) and sensorimotor (with input from premotor cortex and M1) regions of the basal ganglia, suggesting that this region of the cerebellum might be important for the integration of motor and non-motor information (Bostan and Strick, 2010). Consistent with this, in ASD basal ganglia dysfunction has been associated with increased repetitive and stereotyped motor behaviors (e.g., Hollander et al., 2005). Symptom severity in both Tourette syndrome/tic disorder (Stern et al., 2000; Bohlhalter et al., 2006; Lerner et al., 2007; Tobe et al., 2010) and obsessive-compulsive behaviors (Kim et al., 2001; Tobe et al., 2010; Hou et al., 2012), often likened to repetitive and stereotyped motor symptoms in ASD, have been associated with abnormal activation and structure in bilateral Crus I/II. Successful treatment for obsessive compulsive disorder was associated with increased activation in right Crus I (Nabeyama et al., 2008). It is possible that perseverative and repetitive behaviors might be due to loss of modulation of circuits between the posterior cerebellum and basal ganglia.

These results suggest a dissociation between cerebro-cerebellar circuits involved in different types of motor tasks in ASD. Simple motor tasks are associated with abnormal activation in the anterior cerebellum and differences in FC in cerebro-cerebellar somatomotor circuits, whereas reduced activation and FC with cerebro-cerebellar circuits involved in social cognition (right Crus I) are evident during complex motor tasks involving imitation. GM and WM structural differences in the anterior lobe and lobule VIII have been associated with repetitive and stereotyped behaviors in ASD.

### The linguistic cerebellum and cerebro-cerebellar language circuits in ASD

In humans, lobule VII (subdivided into Crus I, Crus II, and VIIB), accounts for the largest proportion of cerebellar volume (Balsters et al., 2010). This considerable volumetric increase compared to phylogenetically older species mirrors the expansion of the frontal lobes, potentially conferring a cognitive advantage (Balsters et al., 2010). Viral-tract tracing studies report anatomical connections between right Crus I and II and BA 46, as well as other language regions of the cerebral cortex (Strick et al., 2009). In typically-developing individuals, right Crus I and II are activated during tasks of language processing, including verbal fluency, grammar, verbal working memory, and language learning tasks (Petersen et al., 1989; Fulbright et al., 1999; Papathanassiou et al., 2000; Mathiak et al., 2002, 2004; Chen and Desmond, 2005a; Booth et al., 2007; Stoodley and Schmahmann, 2010; Sens et al., 2011). The contralateral connections between the cerebellum and cerebral cortex are reflected in the right-lateralization of language-related tasks in the cerebellum, mirroring the left-lateralization of language in the cerebral cortex. Individuals with damage to the right posterior cerebellum can have deficits in both receptive language and expressive language (see Mariën et al., 2014 for review), suggesting that this region of the cerebellum subserves a variety of language functions.

Functional imaging studies in ASD report abnormal activation in these “language” regions of the cerebellum during a variety of language tasks (Harris et al., 2006; Wang et al., 2007; Redcay and Courchesne, 2008; Tesink et al., 2009; Groen et al., 2010). While in typically-developing individuals there was increased activation in right Crus I/II when hearing speech vs. non-speech sounds (Groen et al., 2010), children with ASD had reduced (Wang et al., 2007) or absent activation (Groen et al., 2010) in right Crus I/II in response to vocal stimuli. Reduced activation in right Crus I/II in ASD is often accompanied by hypoactivation in other language-processing regions, including the temporal lobes, medial prefrontal cortex, and Broca's area (Harris et al., 2006; Wang et al., 2007). These data suggest that activation in right Crus I/II and associated cerebro-cerebellar networks is related to basic receptive language processing, and abnormal activation here may be related to impaired communication in ASD.

More complex language processing is also associated with reduced cerebellar activation in ASD, particularly in right Crus I/II. Early PET studies suggested that individuals with ASD had decreased right dentate nucleus activation concomitant with decreased left BA 46 activation during both receptive and expressive language (Müller et al., 1998). During semantic processing (Harris et al., 2006) and processing of semantic anomalies (Tesink et al., 2009; Groen et al., 2010), typically-developing individuals activated right Crus I/II while individuals with ASD showed no statistically significant activation in this region. These data suggest that right Crus I/II might also play a role in semantic discrimination and error-processing in language tasks. Reduced activation here could contribute to the well-documented deficits in language discrimination and semantic processing in ASD (see Groen et al., 2008 for review). These paradigms further suggest that right Crus I/II is hypoactive at multiple stages of language processing in ASD—both initially during listening but also during later semantic processing.

Consistent with functional imaging studies indicating abnormal activation in the posterior cerebellum in ASD, structural differences in these regions are also related to language and fluency impairments in children with ASD. Reduced GM in right Crus I, vermis VI, vermis VIII, and lobule IX correlated with poorer communication skills as measured by standard autism scales (Riva et al., 2013; D'Mello et al., 2015), and reversed asymmetry was observed in lobule VIIIA in language-impaired children with ASD (Hodge et al., 2010). Further, neurochemical markers of reduced neuron density / viability in the right cerebellar hemisphere correlated with fluency deficits in ASD (Kleinhans et al., 2007).

Finally, appropriate recruitment of right Crus I and II might also be important for proper language acquisition and during language learning. In typically-developing infants, GM concentration in right lobule VIIB at 7 months of age predicted receptive language skills at 12 months of age (Deniz Can et al., 2013), and the cerebellum was one of two regions in the brain where GM predicted language skills later in childhood (Deniz Can et al., 2013). The degree of right lateralization in the cerebellum has been associated with stronger core language skills in children (Berl et al., 2014) and increased activation in this area predicted degree of language learning (Pliatsikas et al., 2014a). Studies of second-language acquisition in typically-developing individuals report GM increases bilaterally in lobule VII, which were related to better performance on grammar tasks (Pliatsikas et al., 2014b) and improved fluency (Grogan et al., 2009). Cerebellar activation may also reflect the level of skill acquisition, from novice to expert: Activation in right lobules VI and VII were among the best classifiers of the results of intensive language training, distinguishing trained interpreters from controls (Hervais-Adelman et al., 2015). These findings suggest that the cerebellum may be a crucial neural determinant of language learning.

These data all support a role for the cerebellum (specifically, Crus I and II) in language development and learning. Loss of cerebellar modulatory input on language regions of the cerebral cortex could potentially result in sub-optimal specialization of language circuits, leading to difficulties automatizing language and communication. Consistent with this, lack of functional specialization of cerebral cortical language regions has been well-documented in ASD (e.g., Eyler et al., 2012), and lateralization of language is often abnormal in ASD, with language lateralized to right hemisphere homologs rather than typical left-hemisphere language regions (e.g., Dawson et al., 1982; Escalante-Mead et al., 2003; Takeuchi et al., 2004; Flagg et al., 2005; Knaus et al., 2010; Lindell and Hudry, 2013; Seery et al., 2013). MEG data suggests that while cerebral cortical language representation is originally bilateral in both typically-developing and ASD children, it shifts leftward in typically-developing individuals with age but shifts rightward in ASD (Flagg et al., 2005). The same pattern of abnormal lateralization is noted in the cerebellum. Two- to three-year old typically-developing children recruited right Crus I more strongly than left Crus I (Redcay and Courchesne, 2008), displaying typical contralateral patterns of language activation in the cerebellum. However, age-matched ASD toddlers recruited *left* VI more than right VI (Redcay and Courchesne, 2008). This improper cerebellar lateralization, occurring during a critical period in language development, might result in abnormal specialization of left supratentorial language regions for language.

On the other hand, increased leftward lateralization for language in the cerebellum might allow for compensatory rightward lateralization in the cerebral cortex in ASD (D'Mello et al., 2014). Right cerebral lateralization of language in ASD has been associated with earlier onset of language and better language skills (Joseph et al., 2014). A similar pattern has been noted in cerebellar GM patterns in ASD children with and without early language delay (D'Mello et al., 2014). Both ASD groups showed GM reductions in right Crus I/II, but language-delayed children with ASD also had decreased GM in left Crus I/II (D'Mello et al., 2014). In the face of reduced right Crus I GM, normal left Crus I volumes may enable children with ASD to shift language lateralization to right hemisphere language homologs and compensate for reduced functionality of left cortical language regions. Differences in both right and left Crus I/II might result in abnormal functional specialization of contralateral connected cerebral language homologs as well as right language homologs, leading to language delay (D'Mello et al., 2014).

In addition to well-documented GM reductions in right Crus I/II, ASD children display abnormal structural connectivity between right Crus I/II and the deep cerebellar nuclei. Using MRI tractography, one study found that children with ASD had reduced numbers of Purkinje cell fibers projecting from right Crus I/II of the cerebellar cortex to the right ventral dentate nucleus (Jeong et al., 2014), which then projects to non-motor associations areas of the cerebral cortex, including language regions. In addition, FA was reduced both in short intracerebellar fibers and between right Crus I/II of the cerebellar cortex and the dentate nucleus, which are thought to reflect parallel fiber and Purkinje cell axons, respectively (Catani et al., 2008; Jeong et al., 2014).

In summary, these findings suggest that regions of the cerebellum that interconnect with cerebral cortical language networks could be particularly important in receptive, expressive, and higher-level cognitive aspects of language, possibly due to deficient language learning. Recent resting-state connectivity data suggest that disrupted cerebro-cerebellar connectivity (e.g., Jones et al., 2010) is in marked contrast to intact functional connectivity *within* supratentorial language networks: While functional connectivity between cerebral cortical language areas was intact, language-impaired individuals with ASD displayed decreased rsFC between right Crus I/II and cerebral language regions (Broca's area and Wernicke's area, see Figure 3; Verly et al., 2014).

### The “social” and affective cerebellum and associated cerebro-cerebellar circuits in ASD

Viral tract-tracing and human DTI studies link the posterior cerebellum (particularly Crus I/II, lobule IX, and the posterior vermis) with regions of the cerebral cortex involved in social processing and emotion, providing an anatomical substrate for cerebellar involvement in social cognition and affective regulation (Jissendi et al., 2008; Stoodley and Schmahmann, 2010; Buckner et al., 2011; Sokolov et al., 2012). In typically-developing individuals, cerebellar Crus I/II and lobule IX are functionally connected to the default mode and fronto-parietal networks, and largely overlap with regions of the cerebellum involved in language processing (Stoodley and Schmahmann, 2009; Buckner et al., 2011). These regions of the cerebellum are consistently activated during social paradigms, particularly during abstract mentalizing (Van Overwalle et al., 2014). Crus I/II is engaged during imitation, processing of biological motion, animacy attribution (Jack et al., 2011; Jack and Pelphrey, 2014), and emotional facial processing (Deeley et al., 2007); lobule IX has been found to be activated specifically when healthy individuals broke with social norms (Klucharev et al., 2009). These typical activation patterns suggest that Crus I/II might be important in supporting social processing functions while lobule IX might be involved in signaling social conflict. Both Crus I/II and lobule IX of the cerebellum are functionally connected to the temporoparietal junction, temporal poles, and prefrontal cortex, regions implicated in social cognition in typically-developing individuals (Mars et al., 2012) and which are consistently underactivated in ASD during socially awkward situations (Pantelis et al., 2015). Through these connections, the cerebellum might play a role in modulating supratentorial regions involved in social processing and emotion. As discussed above, damage to the posterior cerebellum can result in sub-optimal regulation of mood and behavior, resulting in affective dysregulation, mood disruptions, and behavioral problems (Schmahmann and Sherman, 1998; Riva and Giorgi, 2000).

These activation patterns in typically-developing individuals are consistent with cerebellar regions where participants with ASD show reduced GM. Structurally, decreased GM in the anterior lobe, right Crus I/II, right lobule VIII, and left lobule IX in ASD have been correlated with increased symptom severity in social interaction (Rojas et al., 2006; D'Mello et al., 2015). Similarly, in DTI data, decreased FA in the anterior cerebellum was correlated with increased social impairment (Cheung et al., 2009). While we have categorized the anterior lobe as broadly motor, the medial portion shows functional connectivity with limbic networks (Buckner et al., 2011), and GM decreases in this region have been shown to correlate with increased social impairment in ASD (D'Mello et al., 2015).

Functional abnormalities in Crus I and II have been related to deficits in imitation and praxis, which are theorized to contribute to social and communication deficits in ASD (Rogers and Pennington, 1991). As mentioned above, during imitation individuals with ASD hypoactivate right Crus I/II and show decreased connectivity between right Crus I/II and supratentorial regions involved in social processing, such as the superior temporal sulcus and superior parietal lobe (Jack and Morris, 2014). Further, deficits in these circuits have been related to impairments on mentalizing tasks (Jack and Morris, 2014), and mentalizing / theory of mind deficits are commonly reported in ASD (e.g., Baron-Cohen, 2000). During mentalizing tasks, typically-developing individuals exhibited greater connectivity between the ventromedial prefrontal cortex and left IV/Crus I in self-mentalizing tasks when compared to mentalizing about others; this FC pattern was absent in ASD (Lombardo et al., 2010). Further, stronger FC between right Crus I and the superior temporal sulcus during mentalizing tasks was associated with better mentalizing abilities in ASD (Jack and Morris, 2014). On a related note, ASD individuals who are classified as highly alexythymic underactivated right VI/Crus I both during processing of pain to the self as well as during empathic pain tasks (Bird et al., 2010).

Crus I/II dysfunction might also contribute to the well-characterized deficits in face-processing in ASD. Activation in left Crus I/II was reported in individuals with ASD during stranger face-processing (Pierce et al., 2004) and during a face-memory task (Koshino et al., 2008), whereas typically-developing participants did not engage this region. During emotional face-processing of happy, sad, disgusted, and fearful faces, ASD individuals showed consistent hypoactivation in bilateral VI/Crus I/II of the cerebellum (Deeley et al., 2007). Unlike other regions of the brain, which were specifically hypoactive only for certain emotions or intensities, bilateral Crus I/II was consistently underactivated in ASD for all face stimuli (emotional faces and neutral faces) (Deeley et al., 2007). This is in marked contrast with the robust right Crus I/II activation in typically-developing individuals during processing and imitation of emotional facial expressions (Leslie et al., 2004; Schutter and van Honk, 2005; Dapretto et al., 2006; Schutter et al., 2009). Further, when attempting to detect irony in faces and prosody, ASD participants underactivated bilateral Crus I/II (Wang et al., 2007) and had fewer responses overall, potentially reflecting difficulty interpreting speaker intent (Wang et al., 2007). Combined with data implicating abnormal Crus I/II activation in language processing, irony, and prosody, abnormal activation in Crus I/II during face processing might further contribute to social impairments in ASD.

In terms of social interaction, children with autism showed abnormal age-related connectivity between the ventral striatum and bilateral lobules VI/Crus I. While typically-developing children showed decreasing rsFC between the cerebellum and ventral striatum with age, children with ASD show aberrant increases in cerebello-striatal connectivity with age (Padmanabhan et al., 2013). The ventral striatum is related to reward learning (Spanagel and Weiss, 1999; Haber, 2011) as well as affective processing (Haber, 2011), and rsFC abnormalities in these circuits could be related to deficits in social interaction in ASD. Consistent with this, some theories of autism suggest that individuals with ASD do not find social interaction rewarding, and are therefore unmotivated to engage in social interaction (e.g., Chevallier et al., 2012).

Connections between the cerebellar vermis and limbic regions of the cerebral cortex might also be relevant to ASD; structural and functional differences in these cerebro-cerebellar loops might be associated with difficulties in a range of affective processing tasks. One of the earliest reported neural differences in ASD was hypoplasia of the posterior cerebellar vermis (Courchesne et al., 1988, 1994a,b), and decreased volume in the posterior vermis inversely correlated with frontal lobe volumes in ASD (Carper and Courchesne, 2000). In typically-developing individuals, the posterior cerebellar vermis is functionally connected to the limbic network (Buckner et al., 2011) and is heavily implicated in affective regulation and emotion (see Schutter and van Honk, 2005; Stoodley and Schmahmann, 2009 for review). In children, damage to the vermis and vermal malformations are associated with affective dysregulation, behavioral deficits, and ASD symptoms (Levisohn et al., 2000; Tavano et al., 2007). Similarly, in ASD reduced GM volume in the anterior vermis and vermis VI correlated with more impaired social interaction scores (D'Mello et al., 2015). Functional MRI studies also report abnormal vermal activation in ASD: Processing of irony was related to decreased activation in medial lobule VIII (Wang et al., 2007), and processing of facial expression resulted in abnormal recruitment of the posterior cerebellar vermis in ASD participants (Critchley et al., 2000).

## Converging findings

**Figure 4:**
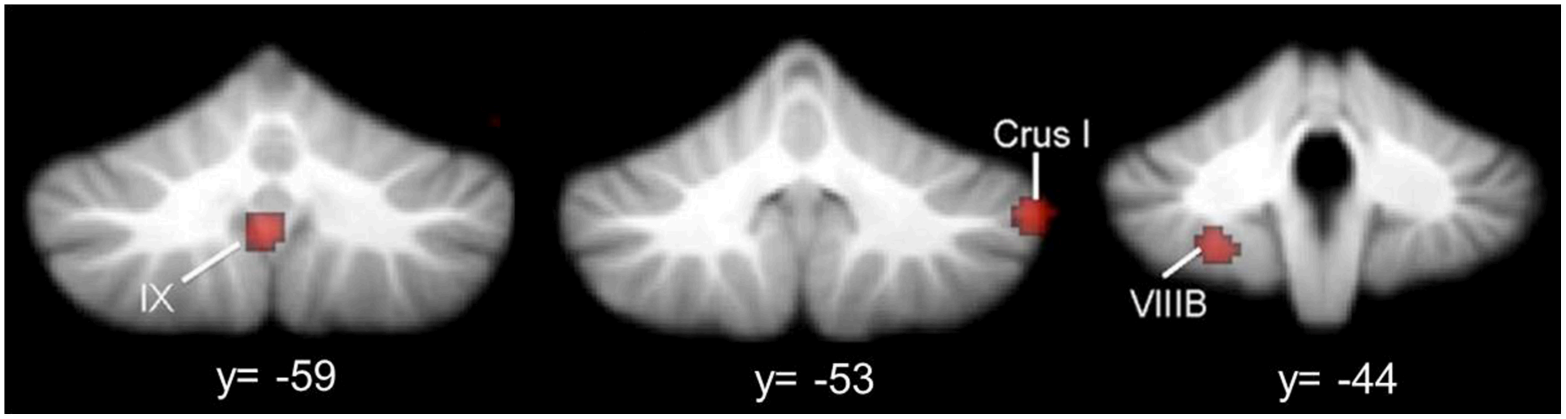
**Cerebellar gray matter reductions in autism**. GM reductions in the cerebellum in ASD, based on a meta-analysis of voxel-based morphometry studies (Stoodley, 2014). Consistent GM reductions are evident in right Crus I, left VIIIB, and midline IX. Figure adapted from Stoodley (2014).

**Figure 5:**
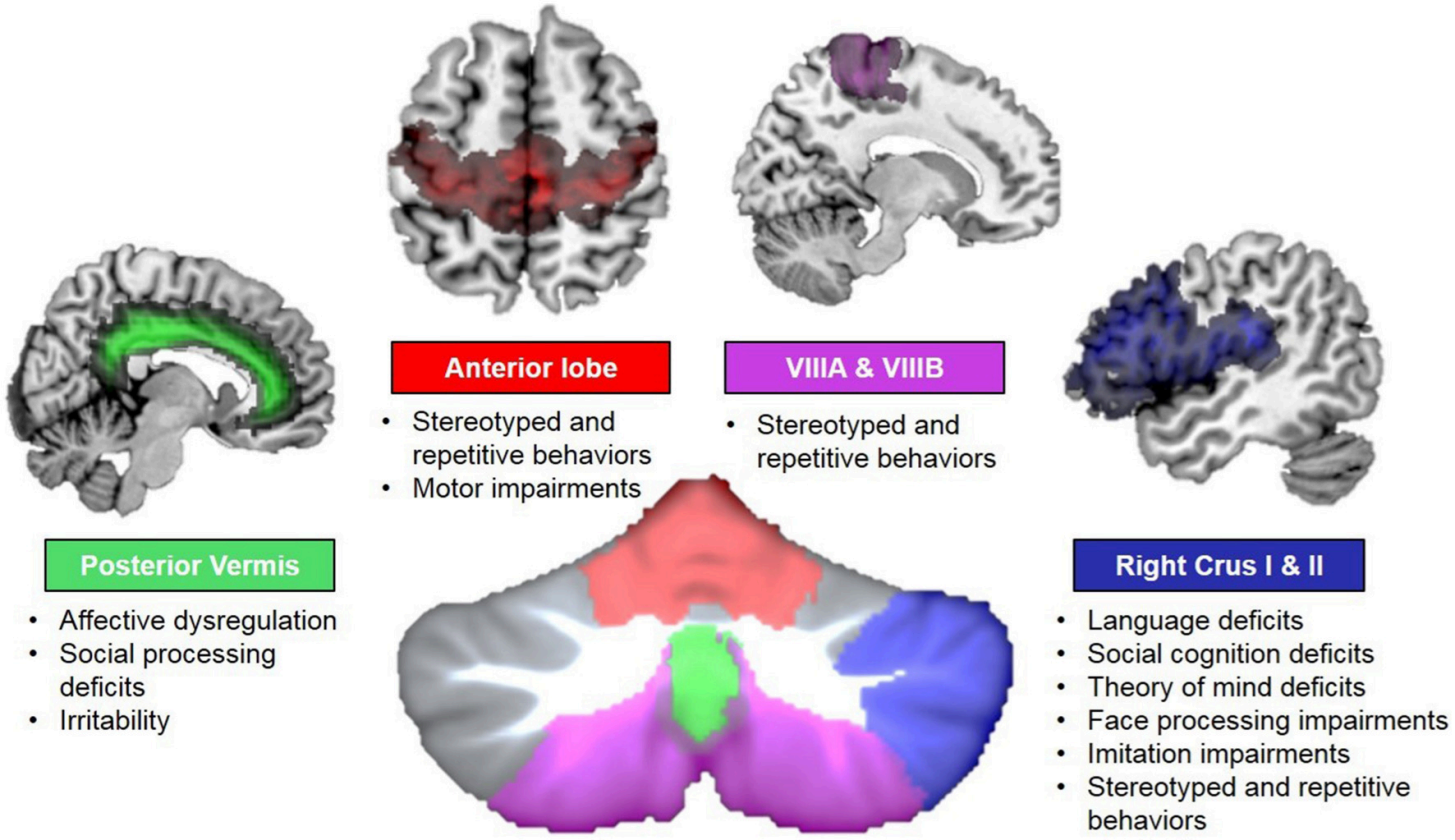
**Cerebro-cerebellar circuits in autism**. Disruptions in specific cerebro-cerebellar circuits could result in different behavioral symptoms of ASD. Colors reflect connectivity of specific cerebellar regions: anterior lobe (red) and lobule VIII (violet) and somatomotor circuits; right Crus I and II (blue) and frontal language areas (among others); and posterior vermis (green) and limbic networks. Behavioral deficits associated with structural and functional disruptions in each circuit are noted.

Based on meta-analyses of structural and functional neuroimaging data, several regions of the cerebellum consistently emerge as abnormal in ASD. Out of 6 whole-brain structural MRI meta-analyses examining the current state of the ASD literature (Stanfield et al., 2008; Cauda et al., 2011; Via et al., 2011; Yu et al., 2011; Stoodley, 2014; DeRamus and Kana, 2015), all but one reported cerebellar differences in ASD (Via et al., 2011; this study used a different approach than the other voxel-based analyses). The most commonly reported differences have been localized to right Crus I, lobule VIII, and lobule IX (Stanfield et al., 2008; Cauda et al., 2011; Yu et al., 2011; Stoodley, 2014; DeRamus and Kana, 2015; Figure 4). These regions, as discussed above, may be associated with specific aspects of the ASD phenotype (Figure 5). Functionally, a meta-analysis of fMRI findings in ASD further supports the relationship between disruption in specific cerebro-cerebellar circuits and task performance, with decreased activation in ASD during motor tasks in the anterior cerebellum, and differences in activation during auditory and language tasks bilaterally in VI and Crus I (Philip et al., 2012).

Abnormal findings in ASD are often right-lateralized, suggesting a specific dysfunction of the right cerebellum and its structural and functional connections with both contralateral and ipsilateral areas of the cerebral cortex (Noonan et al., 2009; Fitzgerald et al., 2015). Within the cerebellum, reduced FA between the right cerebellar cortex and right ventral dentate nucleus was found in over 70% of children with ASD (Jeong et al., 2014). Of note, decreased GM (Rojas et al., 2006; D'Mello et al., 2015) and abnormal activation in right Crus I/II has been related to motor, communication, and social symptoms in ASD, potentially speaking to the role of this region as a biomarker for the “core” ASD diagnosis. This region also shows abnormal structural and functional connectivity in ASD, both locally within the cerebellum (Paakki et al., 2010) and in long-range connections with motor and non-motor supratentorial regions (Noonan et al., 2009; Itahashi et al., 2014, 2015; Jung et al., 2014; Verly et al., 2014; Khan et al., 2015).

These converging findings emerge in the context of the well-documented heterogeneity in ASD, including inconsistencies in the direction of the GM differences in ASD (some studies report increases while others report decreases). Variations in age, IQ, and behavioral phenotypes of participants could contribute to such inconsistencies. Two recent meta-analyses suggest that certain regional cerebellar differences in ASD might be related to age and/or IQ of participants: DeRamus and Kana (2015) reported that decreased GM in vermal IX/VIIIB occurred in 6–16 year olds with ASD, but was not present in the 18–52 age group; Stanfield et al. (2008) also reported that decreased posterior vermal volumes in ASD become less apparent with increasing age, and, in older groups, differences here were less evident when groups were matched for IQ. Further, different behavioral phenotypes within ASD may also contribute to divergent structural findings in ASD. For example, we have found that ASD children with a history of early language delay show different GM patterns within the cerebellum, with decreased left Crus I/II volume specific to the children with early language delay (D'Mello et al., 2014).

## What is the specific contribution of cerebellar processing during development?

Converging data suggest that the cerebellum may play an important role in the developing brain, and that dysfunction in specific cerebellar regions could lead to developmental disorders such as ASD. That said, it is clear that ASD results from dysfunction in multiple regions of the brain, and not only the cerebellum, which leads to the question: What is the specific contribution of the cerebellum to ASD?

In the motor domain, the cerebellum is involved in modulating and automatizing movement in order to optimize performance in a given context (Ito, 2002); transcranial magnetic stimulation of the cerebellum modulates activation patterns in the primary motor cortex (Galea et al., 2011), confirming that altering cerebellar activity has knock-on effects on the regions of the cerebral cortex to which it projects. Information sent from the cerebral cortex and spinal cord is used to create and train internal models of behavior, enabling optimization and prediction of future behavior (Ito, 2008). It is important to note that damage to the cerebellum does not result in complete loss of function (Schmahmann, 1991). For example, classic motor symptoms following cerebellar damage include not paralysis, but rather erroneous and poorly calibrated dysmetric movement. It has been suggested that the cerebellum plays a similar modulatory role in cognition and affect (see Ito, 2008). Akin to the motor symptoms following cerebellar damage, damage to the posterior cerebellum does not result in severely impaired cognition, but rather an inability to modulate and optimize cognitive performance (conceptualized as “dysmetria of thought,” see Schmahmann, 1991). For example, posterior cerebellar damage can result in agrammatism or semantic fluency, but not complete loss of language (Schmahmann and Sherman, 1998).

The process of building and optimizing internal models is directly associated with the role of the cerebellum in implicit learning and skill acquisition. The cerebellum is thought to be maximally involved in initial motor skill learning, while other neural structures (including cortico-striatal pathways and primary motor cortex) are more involved in the retention of learned motor behaviors as a result of extended practice (Doyon et al., 2002; Galea et al., 2011). The same may be true in cognitive tasks, such as working memory: in a study of verbal working memory, right Crus I/II and the contralateral inferior frontal gyrus were maximally activated during the encoding portion of a letter-matching task, while lobule VIII and the posterior parietal cortex were activated during the maintenance phase; no cerebellar activation was associated with subsequent recall (Chen and Desmond, 2005b). A cerebellar role in implicit/procedural learning and skill acquisition is particularly compelling in the context of development and developmental disorders. Indeed, it has been proposed that while declarative memory and learning mechanisms are relatively intact in developmental disorders including dyslexia, developmental coordination disorder, and ASD, implicit skill acquisition is impaired (Biotteau et al., 2015; Ullman and Pullman, 2015). In our view, implicit learning of different types of information (e.g., literacy vs. motor skills vs. social skills) is supported by different cerebro-cerebellar circuits. This is consistent with the lack of overlap of cerebellar structural gray matter reductions between, for example, developmental dyslexia and autism (see Stoodley, 2014). Therefore, behavioral symptoms characterizing a given developmental disorder should reflect differences in structure and function of specific cerebellar regions (Stoodley, 2015); likewise, disorders sharing similar behavioral deficits may be associated with disruption in overlapping cerebro-cerebellar circuits. For example, Stuttering (a disturbance in motoric aspects of speech) is associated with over-activation in the cerebellar anterior lobe (see Stoodley and Schmahmann, 2015), whereas posterior regions of the cerebellum are associated with communication impairments in ASD. On the other hand, shared symptoms of compulsive/repetitive and stereotyped behaviors in obsessive-compulsive disorder and ASD are both associated with abnormalities in right Crus I/II (Kim et al., 2001; Tobe et al., 2010; Hou et al., 2012). The complex behavioral profile of ASD is reflected in the multiple cerebro-cerebellar circuits where structural and functional differences are found, encompassing cerebellar regions involved in movement, language, social cognition, and affective regulation. Disrupted implicit learning specifically affecting the circuits described above could impact the acquisition of motor, communication, and social skills during early development in ASD, leading to long-term deficits in these domains.

### Is the cerebellum involved in “optimization of function” during development?

As mentioned above, the creation of internal models to optimize both cognitive and motor behaviors may be crucial for skill acquisition during typical development. Consistent with this idea, it has been proposed that the integrity of cerebro-cerebellar loops might be especially important earlier, rather than later, during the course of development (Wang et al., 2014), as early cerebellar damage is related to worse outcomes than cerebellar damage in adulthood. For example, during a pivotal period in language development, toddlers aged 1-2 years showed *greater* activation in the anterior vermis as well as bilateral lobule VI of the cerebellum than did older 3 year olds when listening to speech (Redcay et al., 2008). Evidence such as this suggests that cerebellar involvement might be age-dependent—more important earlier in life when cortical networks are first being established, and less important later in life when motor and cognitive behaviors have been appropriately set up in distributed cortical networks. For example, cerebellar processing might support language development by helping to organize cortical regions involved in language, which come on-line later in development and are reliant on appropriate input. In fact, activation in bilateral lobule VI, primarily seen in younger children, showed a negative relationship with expressive language scores, suggesting that decreased activation in this region as language skills develop might reflect a more mature language profile (Redcay et al., 2008).

Given the role of the cerebellum in modulating cerebral cortical activity, cerebro-cerebellar loops might inform early functional specialization of cortical regions. One study examining primary motor cortex in children with ASD found abnormal functional organization of M1 subregions, suggesting a lack and/or delay of functional specialization in this region (Nebel et al., 2014). Abnormal connectivity between the cerebellum and cerebral motor regions might result in sub-optimal automatization and modulation of motor behaviors, and might also be related to delayed acquisition of gestures important for social interaction and communication (Mostofsky et al., 2009). Similarly, abnormal connectivity between the cerebellum and cerebral cortical regions involved in language (Verly et al., 2014) could lead to atypical organization of language networks in ASD (Eyler et al., 2012; Verly et al., 2014), and be associated with delayed language acquisition in ASD. Finally, regions of the cerebellum showing abnormal structure and functional activation in ASD form circuits with cerebral cortices underpinning social cognition (e.g., superior temporal sulcus). It is possible, therefore, that early cerebellar dysfunction can result in sub-optimal specialization of functional networks related to core ASD symptoms of social and communication deficits and repetitive and stereotyped behaviors. Increased repetitive or stereotyped behaviors, atonal or agrammatical language, and impairments in social interaction all reflect not loss of function, but loss of *optimal* function.

### Is the cerebellum involved in “optimization of structure”?

In addition to this proposed role for the cerebellum in optimization of function during the course of development, the cerebellum might also be involved in the optimization of structure. Optimization of structure relies on functional activation: myelination and pruning in the developing brain are known to be activity-dependent, shaping the specialization of structural networks. Longitudinal development of the cerebellum mirrors that of the cerebral cortex, with phylogenetically newer regions, such as the posterolateral cerebellum, reaching peak maturity later in development (Tiemeier et al., 2010). It is possible that these reciprocally-connected regions are developing in concert, such that cerebellar dysfunction has knock-on effects on cerebral cortical development. Consistent with the idea that the integrity of cerebro-cerebellar loops might be especially important for early cerebral cortical development (Wang et al., 2014), damage to the cerebellum early in life can affect the growth and structure of the cerebral cortical regions to which it projects. Infants sustaining cerebellar hemorrhages after birth later had reduced gray matter volume in the contralateral cerebral hemisphere (Limperopoulos et al., 2010, 2012), accompanied by long-term behavioral deficits in movement, language, and general cognition (Limperopoulos et al., 2007). In ASD, developmental differences in cerebellar structure may lead to improper processing of information that is then sent to the cerebral cortex, potentially impacting the activity-dependent structural specialization of the regions of the cerebral cortex to which these cerebellar regions project. Crucially, there is a specificity to the regional findings within the cerebellum in ASD, suggesting that impairments in specific cerebro-cerebellar loops might result in suboptimal structural development in cerebral regions involved in motor, language, and social function, resulting in long-term behavioral deficits.

### Caveats and limitations

While there is robust evidence of cerebellar structural and functional differences in ASD, multiple regions of the brain show abnormalities in this complex disorder. While in our description of abnormal cerebro-cerebellar circuits in ASD we have focused on the cerebellum as the potential “starting point,” it is possible that the differences in cerebellar structure and function result from an initial developmental abnormality elsewhere in the brain. While genetic, animal, clinical, and post-mortem studies suggest that cerebellar differences arise very early in pre-natal development in ASD, and that cerebellar abnormalities alone are sufficient to produce ASD symptoms, it is possible that poor cerebellar information processing is a result of impoverished information reaching the cerebellum. Future studies, described below, should help to clarify if ASD can truly be considered a “disorder of the cerebellum” (Rogers et al., 2013).

## Conclusions and future directions

Anatomical, neuroimaging, and animal work suggest that the cerebellum is one of the most common sites of abnormality in ASD (Fatemi et al., 2012), and cerebro-cerebellar circuits provide a critical anatomical substrate by which cerebellar dysfunction impacts core ASD symptoms. Crucially, damage to the cerebellum can directly lead to an ASD diagnosis in a way that damage to other regions commonly implicated in ASD cannot, including the prefrontal cortex, basal ganglia, and parietal cortex (Riva and Giorgi, 2000; Limperopoulos et al., 2007; Wang et al., 2014). The localization of gray matter and white matter differences in the cerebellum in ASD suggest disruption of specific cerebro-cerebellar circuits involved in movement, language, social cognition, and affective regulation (Figure 5). We suggest that developmental abnormalities in the cerebellum could exert long-term effects via lack of appropriate modulation of the cerebral cortex, impacting the optimization of both structure and function.

Based on these data, future studies should not exclude the cerebellum in analyses of structural and functional differences in ASD. Further, to better characterize cerebellar abnormalities in ASD, neuroimaging investigations should aim to localize cerebellar differences to specific subregions. In addition, the location of these abnormalities must be considered in the context of broader cerebro-cerebellar circuits, in order to better understand the relationship between these differences and specific ASD symptoms. Recent technological advances in high resolution imaging of the cerebellum (e.g., Dell'Acqua et al., 2013) might provide improved understanding of the microstructural organization of cerebro-cerebellar circuits in ASD. In animal studies, disruption of specific cerebellar regions at particular time points could inform our understanding of the developmental relationships between the cerebellum and the cerebral cortex, and further characterize ASD-like behaviors following cerebellar damage. Similarly, human clinical lesion studies throughout the lifespan and longitudinal study designs are necessary to establish the developmental effects of cerebellar damage on optimization of structure and function in the cerebral cortex. Finally, the investigation of the role of the cerebellum in ASD should include tasks that tap not only cerebellar motor function, but also the broader role of the cerebellum in language and social interaction, consistent with our modern understanding of cerebellar function.

## Funding

This work was supported by the National Institutes of Health under award number R15MH106957.

### Conflict of interest statement

The authors declare that the research was conducted in the absence of any commercial or financial relationships that could be construed as a potential conflict of interest.
